# Global Challenges: Water

**DOI:** 10.1002/gch2.1002

**Published:** 2015-09-21

**Authors:** David Butler

**Affiliations:** ^1^ Centre for Water Systems University of Exeter Exeter EX4 4QF UK

## Abstract

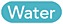

Water is by its very nature paradoxical. On the one hand, it is an abundant global resource at over 1400 million km^3^; on the other hand, it is a scarce commodity with just 0.001% being readily available for human consumption with huge geographical variations even of that small fraction (PWC, 2012). Water is essential to life (survival time is less than a week without water) yet is also life threatening. Contaminated water is the main cause of diarrhoea leading to 4% of all deaths and 5% of health loss to disability worldwide (Water Sanitation Health, 2015). In a 10‐year period to 2009, Europe experienced over 200 major floods, leading to 1126 deaths and the displacement of half a million people (Fitton et al., 2015). Water is unique and non‐substitutable, and yet often taken for granted and poorly valued, especially in the developed nations (http://thevalueofwater.org/). Thankfully, this is gradually changing, and a recent survey of Asian business leaders for the World Economic Forum identified water supply crises as amongst the highest impact risks facing the world today (Climate change and water shortage main concerns at World Economic Forum on East Asia, 2015).

Perhaps the most important global water challenge of all still remains: ensuring access to adequate and equitable drinking water and sanitation for all. Significant progress has been made since the Millennium Declaration in 2000, which laid out eight aspirational goals to be achieved by 2015. In 2015, 91% of the world population has access to an improved drinking water source, compared with 76% in 1990. Globally, 147 countries have met the drinking water target and 95 have met the sanitation target. Worldwide, 2.1 billion people have gained access to improved sanitation. Yet much more needs to be done, 748 million still lack access to improved drinking water, 1.8 billion use a source of drinking water contaminated with faeces and 2.4 billion people do have not have improved sanitation (UN, 2015). The follow‐on, but still draft, Sustainable Development Goals reflect this ongoing challenge with the inclusion of a 2030 target of ensuring the availability and sustainable management of water and sanitation for all (Sustainable Development Knowledge Platform, 2015).

The influence of global climate change is perhaps felt most keenly by water. Both observational records and climate projections show that freshwater resources are particularly vulnerable and have the potential to be strongly impacted (Bates et al., 2008). Climate change also has the potential to increase intense rainfall and raise sea levels, both of which increase the risk of flooding. It has been estimated that flood damage across Europe could increase by 200% by the end of the century (Fitton et al., 2015). The traditional stationarity concept that past hydrological experience provides a good guide to the future no longer holds, and this brings with it enormous challenges to managing water into the future (Milly et al., 2008).

The importance of water goes beyond its links even with health and climate change. Water is intimately intertwined with other key global resources such as food and energy. Globally, 70% of water withdrawals are for the agricultural sector and 15% for energy production, with the latter set to increase to 20% by 2035 (PWC, 2012; International Energy Agency, 2012). Water, energy and food have an almost symbiotic relationship. Simply put: water is needed to generate energy and energy is needed to supply water, water is needed to grow food and food transports (virtual) water, energy is needed to produce food and food can be used to produce energy. A distortion in any one sector has important influences on the others, and a growing world population results in increasing demand for all.

Water is a global challenge, yet its benefits and impacts are very much expressed and tackled at the local level. Even the richest and best‐prepared countries can be tested. As I write this editorial, California in the USA is suffering from a 4‐year drought prompting the state's governor to mandate reductions in water use by residents and public agencies (but not yet agriculture). This has already provoked significant innovation, with Orange County developing a portfolio of water resource strategies including the Water Factory 21 aquifer replenishment scheme using treated municipal wastewater (Force, 2015).

Our ambition for this journal is bold as we wish to address and confront any and all of these global water issues and more. Our scope is also wide, but our modus operandi is different in at least two ways. Firstly, we are challenge‐focused, not discipline‐focused so we welcome contributions from any and all disciplines, and especially from multidisciplinary perspectives. In doing so, we want to stimulate debate between sectors and encourage collaboration. Secondly, we are adamant that the impact of your work outside narrow disciplinary circles should be explicitly highlighted as part of the published paper. In doing so, we intend to help bridge the gap between research and policy to place the work into context for a broad‐based group of stakeholders and to make a real contribution towards addressing the many pressing global challenges of our time.
